# New technologies in screening for breast cancer: a systematic review of their accuracy

**DOI:** 10.1038/sj.bjc.6601836

**Published:** 2004-04-27

**Authors:** L Irwig, N Houssami, C van Vliet

**Affiliations:** 1 Screening and Test Evaluation Program (STEP), School of Public Health, A27, University of Sydney, NSW 2006, Australia

**Keywords:** mass screening, breast cancer, sensitivity and specificity, radiography

## Abstract

We systematically reviewed the literature on the accuracy of new technologies proposed for breast cancer screening. Four potential tests were identified (ultrasound, magnetic resonance imaging (MRI), full-field digital mammography (FFDM), and computer-aided detection (CAD)) for which primary studies met quality and applicability criteria and provided adequate data on test accuracy. These technologies have been assessed in cross-sectional studies of test accuracy where the new test is compared to mammography. Ultrasound, used as an adjunct to mammography in women with radiologically dense breasts, detects additional cancers and causes additional false positives. Magnetic resonance imaging may have a better sensitivity (but lower specificity) than mammography in selected high-risk women, but studies of this technology included small number of cancers. Computer-aided detection may enhance the sensitivity of mammography and warrants further evaluation in large prospective trials. One study of FFDM suggests that it may identify some cancers not identified on conventional mammography and may result in a lower recall rate. The evidence is currently insufficient to support the use of any of these new technologies in population screening, but would support further evaluation.

Despite recent controversy surrounding the efficacy of mammographic screening, it remains the only screening test for breast cancer that has been extensively evaluated in randomised controlled trials (RCTs) and shown to reduce breast cancer mortality (IARC, 2002; Nystrom *et al*, 2002). Since evidence exists that early detection reduces mortality from breast cancer, it is reasonable to evaluate a new screening test by assessing its effect on early detection of breast cancer. While RCTs examining mortality as an outcome are the gold standard, studies assessing new tests are commonly evaluated using surrogate measures as indicators of early breast cancer detection. These surrogate measures may be measurable at the time of screening or require follow-up. Immediate indicators include the cancer detection rate and the size, stage, and nodal status of cancers detected. The measure requiring follow-up is the interval cancer rate. Ascertainment of interval breast cancers poses a number of challenges that include identification (requiring linkage to cancer registries), standardisation, and validation of review and categorisation methods, as well as access to the films taken at diagnosis. Assessing whether tests differ in their interval cancer rate is best assessed by randomising people to the different tests. In designs in which women are assessed by both tests, women in whom cancers are detected by either test would obviously be identified and treated; interval cancers that arise thereafter would be those missed by both tests. To assess whether interval cancer rates differ between the tests is therefore best assessed by randomising women to the different tests. However, all of the immediately measurable surrogates can be assessed in cross-sectional analytic studies of people in whom both of the different tests have been carried out.

New screening methods can be evaluated relative to the proven screening test (screening mammography being the comparator test for breast cancer) as a *replacement* for the comparator or as an *incremental* (additional) method, whereby the new test would only be carried out in those where the comparator test is negative. To allow estimation of both replacement and incremental accuracy, the basic study design is to screen women independently by both mammography and the new test, and assess how many cancers and false positives are detected by one test and not the other. If the objective is to assess incremental accuracy only (additional cancers detected, as well as additional false positives caused by use of the new test), this can be carried out by re-examining only those who were negative on mammography and can be carried out with or without knowledge of the mammography result.

Over the past two decades numerous breast imaging tests have been evaluated and used mainly as adjunct diagnostic methods to mammography, and some have been proposed as possible screening tests. Some of these tests, such as computer-aided detection (CAD), are rapidly evolving and continue to be evaluated. Other tests, primarily ultrasound, have an established role in the investigation of symptomatic women and in the evaluation of screen-detected findings. In this paper, we present a systematic review of published work on the accuracy of new technologies that have been proposed for breast cancer screening.

## MATERIALS AND METHODS

To avoid bias, we systematically reviewed the literature using methods that ensure all relevant studies were identified (Appendix A, literature search methods). The quality and applicability of studies were assessed before their findings were examined (Glasziou *et al*, 2001) (Appendix B, strategy for selection of eligible studies). As both sensitivity and specificity are important in the screening context, our selection criteria required studies to have reported data on both sensitivity and specificity (Appendix B). To ensure the applicability of the results to screening, the studies had to have been carried out on women eligible for screening. Studies on symptomatic women cannot be used to infer the accuracy of a new technique for screening, because the objective of testing is different. In clinical settings, the objective is to determine whether an identified clinical abnormality is cancer. In screening, it is to perceive abnormalities that may be found on further testing to be early cancers. Furthermore, the spectrum of disease usually differs in that cancers which present clinically would be expected to be larger and more advanced.

Papers were therefore included only if they evaluated new tests in asymptomatic women, including populations considered at higher risk for breast cancer because of genetic predisposition or those in whom mammography may be less accurate because they are younger or have radiologically dense breast tissue. Very few studies fulfilled these criteria. The remainder of the papers were review articles, were concerned with the development of the test, or evaluated the use of the test in individual cases or as a diagnostic tool in women with a clinically or mammographically detected breast abnormality. Papers on screening were excluded if important technological changes made them no longer relevant. On these grounds, articles on thermography before 1988 were excluded, as were papers on ultrasonography with water baths or frequency probes with a resolution less than 7.5 mHz. We also excluded studies that compared the proposed technology with an unproven screening method, and on this basis one otherwise eligible study of positron emission tomography was excluded (Yasuda *et al*, 2000). Although studies included used histological diagnosis to verify outcomes, very few papers reported data on interval cancers. However, this does not invalidate comparative studies in which both tests (new technology and mammography) are performed in all women since interval cancers will have been missed by both tests. Our review was concerned with the accuracy of new tests or new technologies associated with mammography, and did not include a cost evaluation.

## RESULTS

No eligible papers were found for CT scanning, magnetic resonance spectroscopy (MRS), scintimammography, electrical impedance, infrared spectroscopy, light scanning, positron emission tomography, or thermography. Eligible studies were identified for ultrasound (Kolb *et al*, 1998; Buchberger *et al*, 1999; O'Driscol *et al*, 2001; Warner *et al*, 2001; Hou *et al*, 2002) magnetic resonance imaging (MRI) (Kuhl *et al*, 2000; Tilanus-Linthorst *et al*, 2000a, 2000b; Stoutjesdijk *et al*, 2001; Warner *et al*, 2001), CAD (te Brake *et al*, 1998; Burhenne *et al*, 2000; Birdwell *et al*, 2001; Freer and Ulissey, 2001), and full-field digital mammography (FFDM) (Lewin *et al*, 2001, 2002). For ultrasound, MRI, and CAD, findings are summarised in Tables 1

**Table 1:** US (table summarises published studies on US in screening, including study design, quality features, and results)

**Reference**	**Objective^a^; comparator**	**Population (mean age in years)**	**Test evaluated 1** **– In consecutive screenees 2** **– Independent of comparator**	**No. of breast cancers (total sample size)**	**TPR or sensitivity**	**FPR (based on solid lesions for US)**
Kolb *et al* (1998, 2002)	I only; M+CBE Views: ND Reader: 1–2	Dense breasts and normal M+CBE (54.7)	1–Yes 2–ND for all subjects (Yes for M-negative/US-positive group)	145; 124^b^ (13 547 screens in 5418 women)	Incremental TPR of US=25.5%	Incremental FPR of US=2.4% (biopsy) and 5.3% (biopsy or follow-up)
						
Hou *et al* (2002)	I+R; M+CBE Views: 2 Reader: 1	‘High-risk’ female relatives of breast cancer patients (48.6)	1–ND 2–ND	21 (935)	US: 90.4% M: 52.4% CBE: 33.3% Incremental TPR of US=33.3%	For abnormal screen – biopsy US: 12.9–2.5% M: 6.0–1.6% CBE: 1.8–1.2% Incremental FPR of US=6.9%
						
O'Driscol *et al* (2001)	I + R; M Views and Reader: ND	Moderate-risk (family history) through clinical genetics unit (42)	1–Yes 2–Yes	2^b^ (150)	US: 50% M: 50%	US: 6.0% M: 0.7% (core biopsy rate)
						
Warner *et al* (2001)	I+R; M+CBE Views: 2 Reader: 1	High-risk, BRCA mutation or several family members (43)	1–ND 2–YES	6; 5^b^ (186)	US: 60% M: 40% CBE: 20% Incremental TPR of US=0%	US: 7% M: 1% CBE: 1%
						
Buchberger *et al* (1999)	I+R; M Reader: 1	Dense breasts and normal CBE (49)	1–ND 2–ND	182; 130^b^ (8970)	US: 99% (invasive), 46% (DCIS) M: 73% (invasive), 90% (DCIS)	US: 4% (biopsy) M: ND

US=ultrasound; M=mammography; CBE=clinical breast examination; ND=not described or unclear from paper; TPR=true-positive rate; FPR=false-positive rate. DCIS=ductal carcinoma *in situ*.

a Objective attainable in using the test: R=replacement for comparator, I=incremental to comparator; Views=number of mammography views per breast, Reader=number of mammography readers.

b Number of cancers reported to be invasive cancers.

, 2

**Table 2:** MRI (table summarises published studies on MRI in screening, including study design, quality features, and results)

**Reference**	**Objective^a^; comparator**	**Population (mean age in years)**	**Test evaluated 1** **– In consecutive screenees 2** **– Independent of comparator**	**No. of breast cancers (total sample size)**	**TPR or sensitivity**	**FPR (% requiring biopsy)**
Warner *et al* (2001)	I+R; M+CBE+US Views: 2 Reader: 1	High risk, BRCA or several family members (43)	1 – ND 2 – Yes	6^b^ (196)	M: 33% CBE: 33% US: 60% MRI: 100% Incremental TPR of MRI=33%	M: 1% CBE: 1% US: 7% MRI: 9%
Stoutjesdijk *et al* (2001)	I+R; M Views: 2 Reader: 1	Breast cancer lifetime risk >15% (40)	1 – No 2 – Yes	12 (259 tests on 166 women)	M: 46% MRI: 100% Incremental TPR of MRI=54%	M: 4% MRI: 7%
Tilanus-Linthorst *et al* (2000a, b)	I only; M+CBE Views: 1–2 Reader: 2	High risk (>25%) and >50% breast density (41.5)	1 – Yes 2 – ND	ND–probably between 3–10 (109)	Two cancers detected by MRI but not by M+CBE	ND
Kuhl *et al* (2000)	I+R; M+US Views: 2 Reader: 2	High risk of familial breast cancer (39)	1 – No 2 – Yes	9 (105)	M: 33% US: 33% M+US: 44% MRI: 100%	M: 7% US: 20% MRI: 5%

MRI=magnetic resonance imaging; M=mammography; CBE=clinical breast examination; US=ultrasound; ND=not described or unclear from paper; TPR=true-positive rate; FPR=false-positive rate.

a Objective attainable in using the test: R=replacement for comparator; I=incremental to comparator; Views=number of mammography views per breast, Reader=number of mammography readers.

b Number of cancers reported to be invasive cancers.

and 3

**Table 3:** CAD (table summarises published studies on CAD in screening, including study design, quality features, and results)

**Reference**	**Objective^a^; comparator**	**Population (mean age in years)**	**Test evaluated 1** **– In consecutive screenees 2 – Independent of comparator**	**No. of breast cancers (total sample size)**	**TPR or Sensitivity**	**FPR (based on recall rate)**
Birdwell *et al* (2001) Burhenne *et al* (2000)	I only; M Views: 2 Reader: 1	Average-risk. CAD assessed on prior M in women later found to have breast cancer	1–No 2–No (yes for non-cancers)	542 (14 500)	M: 79% Incremental TPR of CAD=16%	FPR from before after study M: 8.3% CAD: 7.6%
Freer and Ulissey (2001)	I only; M Views: 2 Reader: 1	Average risk	1–Yes 2–No	49 (12 860)	M: 83.7% Incremental TPR of CAD=16.3%	M: 6.5% CAD: 7.7% Incremental FPR of CAD=1.2%
te Brake *et al* (1998)	I only; M Views: 1 Reader: 2	Women subsequently shown to have breast cancer	1–No 2–No	65 (207)	Incremental true positives reported (difficult to quantify)	Incremental false positives reported (difficult to quantify)

CAD=computer-aided diagnosis; M=mammography; ND=not described or unclear from paper; TPR=true-positive rate; FPR=false-positive rate.

a Objective attainable in using the test: R=replacement for comparator, I=incremental to comparator; Views=number of mammography views per breast, Reader=number of mammography readers.

. There was no evidence originating from RCTs.

Ultrasound (Table 1) has been evaluated primarily in younger women who have mammographically dense breast tissue or are considered to be at elevated risk of breast cancer. The five studies included over 350 cancers. The results suggest that ultrasound may be more sensitive but less specific than mammography in these women. Ultrasound used as an additional test to mammography detects additional cancers, but also increases the false-positive rate.

Magnetic resonance imaging (Table 2) has been examined in four recent studies, which evaluated the test in women at high risk of cancer (usually on the basis of genetic mutations or a family history of breast cancer). In all studies, the technology was contrast-enhanced MRI and all studies used a dedicated breast coil. There were less than 40 cancers in all studies combined. The results suggest that MRI is more sensitive than mammography in selected populations, but may also have a lower specificity. There are currently several trials being conducted in the UK, Europe, and the USA to assess the role of MRI in breast screening (UK MRI Breast Screening Study Advisory Group, 2000), (http://www.acrin.org/current_p rotocols.html. Accessed 5/12/03).

Computer-aided detection is essentially a tool to ‘prompt’ the radiologist to look at potential abnormalities on digitised mammograms and is complementary technology to mammography. In the screening context, it is potentially equal to another ‘read’ (that is, one reader plus CAD may potentially replace two readers). CAD (Table 3) has been assessed in several studies with over 650 cancers. However, only one of these studies was prospectively conducted (Freer and Ulissey, 2001). All of the studies examined the incremental value of CAD and showed improved sensitivity; the evidence on specificity is conflicting. It is not clear to what extent the improvement compares to other manoeuvres, such as having a second film reader.

Full-field digital mammography is a new mammography technology that uses a digital receptor instead of the conventional screen film, resulting in a computer-generated image. Full-field digital mammography has the potential to improve image resolution and image processing and display techniques relative to conventional mammography. Full-field digital mammography has been examined in one study, which screened 4489 average-risk women 40 years and older (Lewin *et al*, 2001, 2002). In this study, 42 invasive cancers were detected (and four interval cancers were identified at 12 months). Full-field digital mammography was found to have a lower overall sensitivity (64.3%) than conventional mammography (78.6%). However, despite having a lower overall sensitivity than conventional mammography, FFDM does result in an incremental gain in sensitivity of 21.4% (that is, it will identify additional cancers that are not identified on conventional mammography). Full-field digital mammography was reported to have a recall rate of 11.8%, which was significantly lower (*P*< 0.001) than the recall rate of conventional mammography (14.9%).

## DISCUSSION

New technologies proposed for breast cancer detection have not been evaluated in RCTs that examine the reduction of the interval cancer rate as indicators of early breast cancer detection. These new technologies have been assessed in cross-sectional analytic studies of test sensitivity and specificity, where the new test is compared to mammography. None of the tests evaluated consistently showed sufficient accuracy in high-quality studies to support their use in population screening. The conduct and reporting of the studies were limited, and the populations were generally too small to allow adequate precision in critical measures, such as test sensitivity. Most of the eligible studies identified in our review did not provide data on interval cancers, and although this is required for an estimation of test sensitivity, it does not invalidate data presented on the difference in sensitivity of the two tests since interval cancers will have been missed by both the new and the comparator test (Chock *et al*, 1997).

Ultrasound may increase the sensitivity of screening if used as an adjunct to mammography for women shown to have radiologically dense breasts, but is likely to increase substantially the number of women requiring biopsy for benign findings. The data from the ultrasound studies are far from conclusive, but does suggest that the potential for ultrasound to replace mammography in screening selected populations warrants further investigation. In addition, ultrasound is highly operator dependent, and the findings from the reported studies may not be applicable in a broader screening context. Magnetic resonance imaging has not been evaluated as a screening test in unselected populations, and its potential role in screening (if any) is in women at high risk of breast cancer.

Based on a limited number of studies, FFDM and CAD show an incremental improvement in sensitivity relative to conventional mammography. However, incremental improvement in sensitivity will not necessarily translate into an absolute benefit, since the new technology may be selectively detecting cancers that are biologically inconsequential. Both FFDM and CAD are currently being evaluated in ongoing studies. Future studies of these two technologies, in particular, should be supported using prospective designs, large samples, and preferably randomised trials using the difference in interval cancers as one of the outcome measures.

Studies that assess new technology before its widespread introduction should be carried out in consecutive women eligible for screening. Cancer is likely to be detected in only a few percent of women screened. Therefore, the confidence intervals for estimates of the sensitivity of new tests, or for differences in sensitivity between new and existing tests, will require screening tens of thousands of women, to detect at least 100 cancers. Future studies should therefore be designed to have adequate sample sizes, for example as is being carried out in the digital mammography screening trial, which aims to recruit 49 500 asymptomatic women presenting for screening (http://www.cancer.gov/dmist and http://www.acrin.org/current_p rotocols.html Accessed 5/12/03). Studies of new tests in any context, should conform to high standards of conduct and reporting (Bossuyt *et al*, 2003), should clarify whether the new test is being evaluated as a replacement or as an additional test, and should use the appropriate study design to assess the intended use of the test. Studies evaluating new technologies in breast cancer screening, relative to mammography, should provide information on the number of views and the number of readers for both the new technology and mammography, and should assess the effect of increasing the number of views or readers.

Although some of the proposed new screening tests show promise, there is a need for larger and better quality of studies of new technology, starting soon after it is introduced to allow concurrent evaluation and implementation (Hunink and Krestin, 2002). As new technology often changes rapidly, it might seem appropriate to leave evaluation until the new technology is ‘stable’. However, methods can be used to assess changes and developments of new technologies as part of the study design to allow early evaluation of new technologies (Hunink and Krestin, 2002). Studies that compare two screening tests or technologies in the same women can provide data on whether new tests do better than the existing ones in detecting more cancers. Before widespread implementation, those tests that show promise in cross-sectional analytic studies should then be evaluated in large randomised trials to ensure that they also reduce the interval cancer rate.

## Appendix A

### LITERATURE SEARCH

MEDLINE was searched from 1966 to December 2002 using the following methods: explode breast neoplasms (all subheadings); explode sensitivity and specificity (all subheadings); explode mass screening (all subheadings); combine previous steps using the Boolean term ‘or’; search term(s) for the specific test of interest and combining using the Boolean term ‘or; combine all previous steps using the Boolean term ‘and’. Searching for specific tests using search term(s) included: CAD; CT scan (tomography); electrical impedance; FFDM; digital mammography; scintimammography (Tc 99m sestamibi, mammoscintigraphy); MRI; MRS; optical mammography (transillumination); PET; spectroscopy; thermography; and ultrasound (sonography).

The search was extended by examining references given in relevant primary studies and review articles, contact with content experts, and targeted further MEDLINE searches, for example on authors of earlier studies. MEDLINE searching identified 649 papers, and our overall search identified more than 1750 papers.

## Appendix B

### STRATEGY FOR SELECTION OF ELIGIBLE STUDIES

The titles and abstracts of all papers were evaluated by one author (CVV) and those possibly relevant were then assessed for final selection by one of the two reviewing authors (reviewing authors: LI or NH). Studies that included the largest number of cancers or those for which it was difficult to extract the information were in addition independently assessed by one of the two reviewers (LI or NH), and differences between the reviewers were resolved by discussion and consensus.

The following information was examined to determine eligible studies, with assessment of quality and applicability criteria *prior* to the extraction of study results:

- Quality (consecutively attending screenees; screening tests applied to all participants; screening tests assessed independent of each other).
- Objective and applicability (Was the study designed to explore the value of the new screening test as a replacement test or to assess its incremental accuracy to existing screening test? Was the study restricted to special groups such as women with high breast cancer risk or dense breast tissue on mammography? To what was the replacement or incremental value of the new test being compared, including descriptive information on number of mammography views and number of readers?).
- Results (number of cancers and total population size; true-positive rate (TPR or sensitivity) and false-positive rate (FPR, 1 – specificity), or as a proxy the test positivity rate or biopsy rate) in the overall screened population. Studies were excluded if they did not report any measure of TPR or FPR.
